# Marine Geo-Polymer Cement Treated with Seawater, Alkaline Activators, Recycled Particles from Paste, and Recycled Particles from Glass

**DOI:** 10.3390/ma17225527

**Published:** 2024-11-13

**Authors:** Xiaoyang Chen, Yajun Wang, Tao Yang, Yang Bai

**Affiliations:** School of Maritime and Civil Engineering, Zhejiang Ocean University, Zhoushan 316022, China; chenxiaoyang@zjou.edu.cn (X.C.); yangtao@zjou.edu.cn (T.Y.); baiyang@zjou.edu.cn (Y.B.)

**Keywords:** marine geo-polymer cement, recycled particle from paste, recycled particle from glass, alkaline activators, carbon fixation, strength, perseverance, microstructure

## Abstract

This study aims to develop the marine geo-polymer cement that was produced with seawater, recycled particles from paste, recycled particles from glass, and alkaline activators, including NaOH or Na_2_O·3.3SiO_2_. The physicochemical properties and strength of MGPC were investigated with a Uniaxial Compression Test, Particle Size Analysis, Energy Dispersive Spectrometer, X-ray Diffraction, and Thermal-field Emission Scanning Electron Microscopy. The results indicated that the main hydration products in MGPC were calcium carbonate (CaCO_3_), silica (SiO_2_), sodium aluminosilicate hydrate (Na_2_O·Al_2_O_3_·xSiO_2_·2H_2_O, N-A-S-H), and aluminum calcium silicate hydrate (CaO·Al_2_O_3_·2SiO_2_·4H_2_O, C-A-S-H). The calcium carboaluminate (3CaO·Al_2_O_3_·CaCO_3_·32H_2_O, CO_3_-AFm) in MGPC was converted into CaCO_3_ and Friedel’s salt (3CaO·Al_2_O_3_·CaCl_2_·10H_2_O), which prompted the carbon sequestration. The microstructure of MGPC prepared using Na_2_O·3.3SiO_2_ was based on RPG as the matrix, with N-A-S-H, C-A-S-H, and fibrous AFt growing on the periphery. This structure reduces the impact of the alkali–silica reaction on the material and improves its compressive strength. Therefore, the MGPC developed in this study shows the exact benefits of freshwater and natural minerals saving, carbon sequestration, and damage resistance.

## 1. Introduction

The traditional method of cement production primarily relies on the combustion of fossil fuels, which not only consumes significant mineral resources but also contributes to global environmental issues, particularly in terms of carbon dioxide emissions. On a global scale, cement production emits at least 1.2 billion tons of carbon dioxide annually, accounting for 7% of total carbon dioxide emissions [1]. Furthermore, large-scale cement production demands considerable amounts of freshwater. The solid waste remaining after a building is abandoned is referred to as construction solid waste, which occupies extensive land areas and has the potential to release harmful metal ions into freshwater ecosystems, worsening environmental pollution.

To mitigate the consumption of freshwater resources, utilizing the Earth’s abundant seawater resources has become a topic of increasing interest. Using seawater as a raw material for concrete production could play a crucial role in conserving freshwater. Notably, the chloride anions (Cl^−^) present in seawater positively influence cement hydration by shortening the cement’s setting time and accelerating its hardening process [2,3,4]. Additionally, higher concentrations of sodium cations (Na^+^) enhance the dissolution of aluminosilicate materials, such as fly ash or metakaolin, thereby facilitating the release of silicon (Si) and aluminum (Al) species into the solution, which are the key components in the concrete structure. These elements play a positive role in the formation of sodium aluminosilicate hydrate (Na_2_O·Al_2_O_3_·xSiO_2_·2H_2_O, N-A-S-H) [5].

Utilizing construction solid waste as a replacement for traditional cement can help alleviate ecological damage. The incorporation of alkaline activators and aggregates positively impacts the performance of geo-polymer cement. Compared with Portland cement, cement-based materials activated by alkaline activators exhibit excellent performance, including better resistance to sulfate corrosion, lower energy consumption during production, reduced carbon emissions, and the ability to use construction solid waste as a precursor [6,7,8]. During the preparation of geo-polymer cement, the use of alkaline activators to restore the hydration capacity of construction solid waste contributes to reducing carbon emissions throughout the geo-polymer cement lifecycle and its overall environmental impact [9].

The Emissions Gap Report (2023) released by the United Nations Environment Programme suggests that carbon dioxide removal should focus on human activities involving the extraction of carbon dioxide from the atmosphere and its long-term storage in geological, terrestrial, or marine reservoirs or in products [10]. Previous studies have indicated that alkaline earth metal cations (Ca^2+^/Mg^2+^) in construction solid waste can facilitate carbon fixation by converting CO_2_ into stable inorganic carbonates [11]. Glass, as an indispensable part of human life, has become an important source of urban household waste due to its short lifespan and high consumption. According to statistics, the output of waste glass in China in 2022 was 24.327 million tons, with a recovery volume of 8.5 million tons and a recovery rate of 34.94% [12]. On a global scale, the glass manufacturing industry emits at least 86 million tons of CO_2_ annually, accounting for 2% of the total carbon dioxide emissions. However, the quality or purity of glass does not change during recycling, so the CO_2_ generated during glass production can be offset by recycling waste glass [13]. Therefore, the recycling and utilization of glass had significant value for carbon neutrality. According to reports, fine glass powder has sufficient volcanic ash properties, and concrete made by partially replacing cement with glass powder exhibits comparable performance to fly ash-modified concrete [14,15,16,17]. According to previous reports, incorporating waste glass powder into alkali-activated cement-based materials can enhance workability, thermal conductivity, and sulfate resistance [18]. However, the potential expansion from the alkali–silica reaction may adversely affect its strength. Although using waste glass for glass recycling can reduce the use of mineral resources, calcination still increases CO_2_ emissions. Utilizing waste glass in the preparation of concrete materials not only provides an additional recycling option for waste glass but also helps reduce carbon dioxide emissions.

Currently, the overall recycling process of construction solid waste remains at a relatively low level, primarily being used as a substitute for natural aggregates, while the potential of construction solid waste has not yet been fully realized [19,20]. Upon processing discarded concrete, it was discovered that its main components are limestone and trace amounts of silica. Due to its high stability and favorable chemical properties, construction solid waste can serve as a calcium source to replace natural limestone or partially replace silica in the production of recycled cement concrete. The resulting product demonstrates properties similar to those of traditional cement concrete [21,22].

Experimental designs involving alkaline activator-activated construction solid waste using construction solid waste, hydrated sodium silicate, and sodium hydroxide as raw materials revealed that the performance of the resulting materials primarily depends on the type of alkaline activator used [23,24,25]. Construction solid waste activated by alkaline activators exhibited excellent compressive strength, and multiple studies have shown that the application of alkaline activator-activated construction solid waste in the production of renewable materials is both viable and effective [26,27].

The environmental pollution and resource consumption resulting from cement production and usage have raised significant concerns about sustainability. Replacing cement partially or entirely with construction solid waste, adding waste glass to produce geo-polymer cement through chemical processes, and using seawater instead of the freshwater typically required for geo-polymer cement preparation presents several advantages, including cost-effectiveness, recyclability, applicability, and reduced carbon emissions. Analyzing the composition of construction solid waste and is essential for developing geo-polymer cement with a robust structure.

Utilizing construction solid waste to prepare recycled particles from paste (RPP) and recycled particles from glass (RPG) can extend the lifespan of materials and reduce environmental damage caused by landfill accumulation. This paper proposes a method for producing marine geo-polymer cement (MGPC) using seawater, alkaline activators, RPP, and RPG. The developed MGPC offers advantages such as low cost, recyclability of construction solid waste, and reduced carbon emissions.

## 2. Materials and Methods

### 2.1. Materials

#### 2.1.1. Information on Seawater and Alkaline Activators

In this study, seawater from the East China Sea was used as a raw material to prepare MGPC. An ICS-5000 ion chromatograph was employed to test and analyze the ion and salt content, as well as the distribution, of the seawater samples mentioned above. In this study, NaOH (NH) and Na_2_O·3.3SiO_2_(NS) were used as raw materials to prepare the alkaline activators, with their specific information detailed in Table 1.

#### 2.1.2. RPP

The RPP used in this study was derived from solid waste from simulated construction. Since most buildings are constructed using cement pastes made with fresh water, this study employed fresh water and P.O 42.5 cement to prepare cement pastes simulating construction solid waste. This study prepared 70.7 mm × 70.7 mm × 70.7 mm cubes using P.O 42.5 type cement and pure water (PW) at a ratio of PW: P.O 42.5 = 1:0.458. The P.O 42.5 cement and PW were mixed evenly in a stirring pot and then poured into an iron mold of the specified dimensions. After pouring was complete, the iron mold was immediately placed in a moist room with a relative humidity greater than 95% and a temperature of 20 ± 2 °C for 24 h.

Once the slurry solidified, the cubes were removed from the mold and continued to cure in the same environment for a total of 35 days. To simulate carbonation under normal usage conditions, this study extended the curing time from 28 days to 35 days, adding an additional 7 days of curing. Uniaxial Compression Tests were then performed on the cubes, and the broken residues were collected. Following testing, each cube’s compressive strength met the M10 grade, which can simulate the solid waste generated after the geo-polymer cement used in projects reaches its service life. The crushed blocks produced from the Uniaxial Compression Test experiment are the simulated construction solid waste studied in this study.

To prepare the RPP from the collected crushed cubes, this study used a hammer crusher and an electrical grinding machine. First, the collected cubes were crushed into granules no larger than 5 mm using a hammer crusher, producing what this study termed granules. Next, these granules were placed in an electrical grinding machine and ground into a powder with a particle size of ≤0.075 mm. Finally, the resulting particle constituted the RPP used in this study.

#### 2.1.3. RPG

The RPG used in this study was obtained from waste glass bottles derived from processed household waste. The waste glass bottles were first crushed into flakes, which were then ground using a precision grinder. The ground glass particles were subsequently sieved using a mesh with a particle size of less than 300 μm, and the resulting particles were collected as the RPG used in this study.

#### 2.1.4. Design and Preparation of MGPC

This study categorized the mixed proportions into NH and NS groups based on the type of alkaline activators used. Given the specific conditions of seawater mixing and curing, this study specifically designed one set of NH as the alkaline activator with a unique seawater content to serve as a control group compared to the other groups. The mix proportions for the other NH and NS groups were established using an equal incremental relationship based on the content of the alkaline activators. Ultimately, this study created 4 sets of ratios using NH as the alkaline activator and 3 sets using NS, resulting in a total of 7 sets of ratios. This design approach allowed for both intra-group and inter-group comparisons while also minimizing study costs and enhancing efficiency. The detailed ratios are listed in Table 2.

In this study, RPP and RPG were prepared according to the ratios shown in the table. Before mixing, the raw materials were divided into solid and liquid components for separate preparation. The solid component was made by thoroughly mixing RPP and RPG, while the liquid component was prepared by adding the alkaline activator to seawater and mixing it well. Once both the solid and liquid components were ready, they were placed into an NJ-160A mortar mixer. The mixer was then started to begin the mixing process. After mixing was completed, the MGPC slurry was poured into a steel mold with dimensions of 30 mm × 30 mm × 30 mm and vibrated for 3 min to ensure proper compaction.

**Figure 1:**
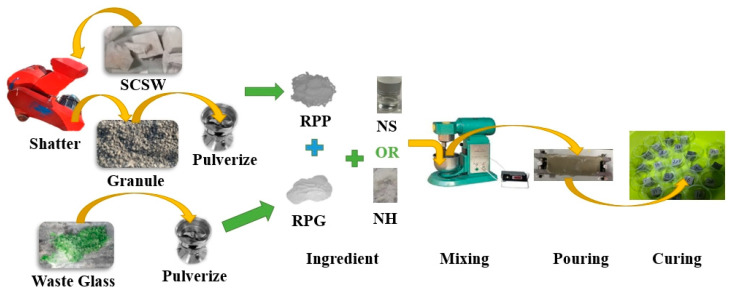
Making method of MGPC (SCSW: simulate construction solid waste). RPP: recycled particles from paste; RPG: recycled particles from glass; NH: NaOH; NS: Na_2_O·3.3SiO_2_; SCSW: simulate construction solid waste.

After pouring, the iron molds were immediately placed in a damp room with a relative humidity greater than 95% and a temperature of 20 ± 2 °C for 24 h. Once the cement paste had been set, the cubes were removed from the molds and completely immersed in seawater for continued curing for 45 days. The reason for choosing 45 days was to facilitate a better reaction with CO_2_ in the environment and to study their performance in a seawater environment. The complete preparation process of MGPC is illustrated in Figure 1.

### 2.2. Methods

This study employed Particle Size Analysis to measure the particle size of recycled construction solid waste (RPP) and recycled glass (RPG). Initially, the finely powdered samples, obtained from crushing and sieving, were placed in a Sieve Shaker to determine the particle size distribution of the materials.

Following the preparation and curing of the MGPC cubes, compressive strength tests were conducted to assess the mechanical properties of the materials. After testing, all fractured specimens were collected, and the area of each cube’s six faces was measured and compared to the cross-sectional area prior to testing to calculate the cross-sectional loss rate. Simultaneously, the mass of the fractured specimens was weighed and compared to the pre-test mass to calculate the mass loss rate. These two metrics were used to evaluate the toughness of the materials comprehensively.

After calculating the cross-sectional and mass loss rates for each mixing ratio, three fractured samples from each mixing ratio were selected for further analysis, including X-ray Diffraction (XRD), Energy Dispersive Spectroscopy (EDS), and Scanning Electron Microscopy (SEM) testing. These analyses provided detailed information regarding the microstructure and chemical composition of the materials, offering scientific insights for the performance evaluation and application of MGPC materials. The equipment used for the testing experiments is listed in Table 3.

## 3. Tests Results and Discussions

### 3.1. Seawater Composition Testing

**Figure 2:**
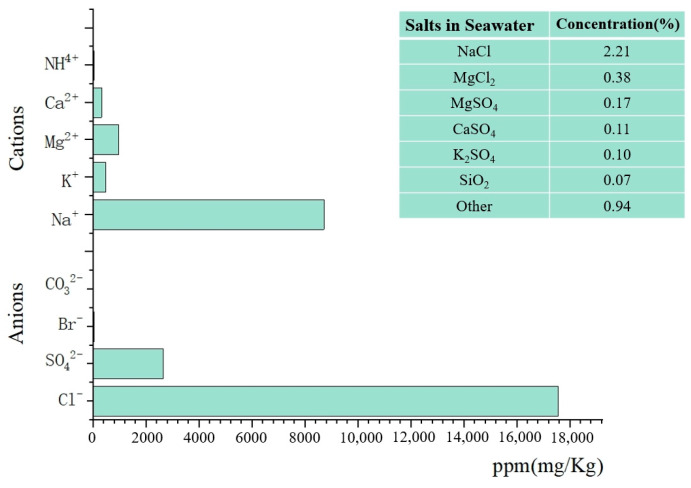
Composition of seawater.

Figure 2 illustrates the composition of the seawater. The analysis revealed that NaCl is the most abundant compound in seawater. The high chloride ion content significantly influences the hydration of cement slurry, as Cl^−^ anions have a stronger reducing ability than OH^−^ anions [28]. As a result, early hydrates lose their alkalinity under the influence of alkali metal salt cations present in seawater, ultimately forming new salts. These salt products undergo further hydration reactions with C_3_A (3CaO·Al_2_O_3_) to produce Friedel’s salts (3CaO·Al_2_O_3_·CaCl_2_·10H_2_O), which are anionic bimetallic layered hydroxides with high reactivity [29]. The interaction between Cl^−^ anions and cement reduces the concentration of Cl^−^ in the surrounding environment, thereby enhancing the early strength of the cementitious material [30].

The addition of seawater increases the concentration of Na⁺ cations in the environment where MGPC is formed, facilitating the dissolution of Al and Si, which positively impacts the production of sodium aluminosilicate hydrate (N-A-S-H). In addition, the presence of Mg^2+^ and K^+^ cations in seawater promotes more complete hydration of MGPC in marine environments. The resulting Mg(OH)_2_ (MH) product effectively inhibits further penetration of corrosive elements such as sulfate attack [31,32]. 

### 3.2. Particle Size Analysis Results and Analysis of RPP and RPG

**Figure 3:**
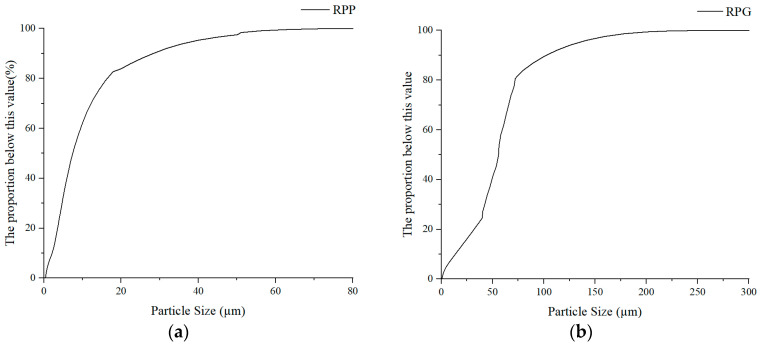
Particle size distribution of RPP and RPG: (**a**) RPP; (**b**) RPG; RPP: recycled particles from paste; RPG: recycled particles from glass.

Calculate the non-uniformity coefficient and curvature coefficient of RPP and RPG based on the obtained particle size distribution; the analysis results are shown in Figure 3 and Table 4. Analysis reveals that each particle size of RPP and RPG had a certain proportion of material particles, indicating that both RPP and RPG had good uniformity. Part of the RPP and RPG will serve as the matrix for hydrate crystallization, playing a nucleation role during the hydration process, further increasing the density of the material, and playing a positive role in adjusting its properties.

RPP generally has a small particle size, resulting in a larger specific surface area. When the specific surface area increases, silicates are more easily activated during the hydration reaction, thereby generating various hydrates, including CaO·SiO_2_·H_2_O (C-S-H) colloids. These hydrates can better improve the pore structure of materials. 

The reactivity of RPG is closely linked to its particle size. RPG particles smaller than 300 μm can exhibit pozzolanic properties, with a significant threshold for pozzolanic activity identified at 75 μm [33,34]. Particles smaller than 75 μm facilitate the dissolution of SiO_2_, increasing the concentration of Si in the environment, which in turn positively influences the formation of silicate colloids [35]. As illustrated in Figure 3b, over 80% of the particles used in this study have a particle size of less than 75 μm. Based on this particle size distribution, it can be concluded that the RPG used in this study possesses pozzolanic properties, which effectively promote the hydration of MGPC.

### 3.3. Uniaxial Compression Test Results and Analysis of MGPC

**Figure 4:**
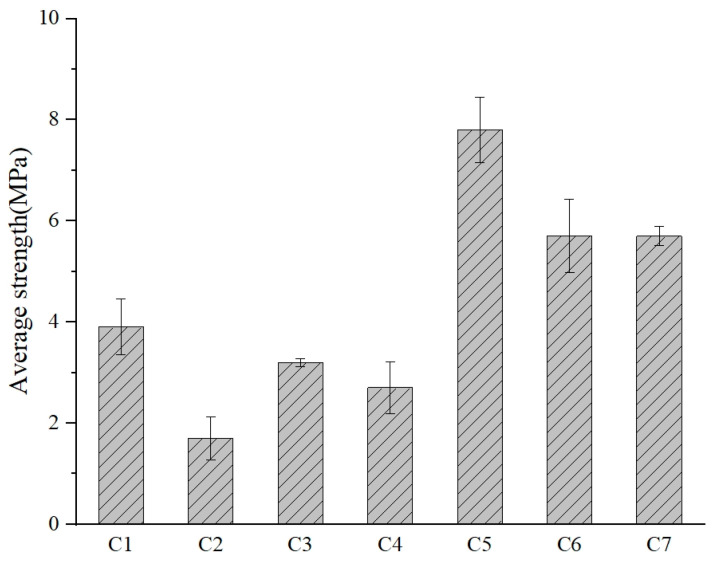
Compressive strength of MGPC cubes at 45 days.

The compressive strength test results are shown in Figure 4. Following the UCT, the mass of each compressed cube was recorded. The mass loss rate was calculated by comparing the remaining mass of the damaged cube to its original mass before testing, using the formula provided in Equation (1).

To evaluate the cross-sectional damage, the remaining areas of the six cross-sections of each cube were measured and compared to their original cross-sectional area prior to compression. The formula used to determine the cross-sectional loss rate is outlined in Equation (2). Detailed results for both the mass loss rate and cross-sectional loss rate are provided in Table 5.

(1) $$\begin{array}{l} \Omega_{m}=\frac{m_{1}}{m_{0}} \end{array}$$

(2) $$\begin{array}{l} \Omega_{a}=\frac{a_{d}}{a_{0}} \end{array}$$

The UCT results indicated that the MGPC with 10.52% NS content exhibited the highest compressive strength compared to other groups. Additionally, all MGPC cubes using NS as an alkaline activator showed higher compressive strength than those using NH as an alkaline activator. However, the compressive strength of the MGPC specimens prepared with either alkaline activator did not demonstrate a trend of variation with changes in alkaline activator concentration. This suggests that the compressive strength of MGPC cubes prepared with seawater is related to the type of alkaline activator used.

The data indicate that, compared to the MGPC cubes with NH, those with NS exhibit higher compressive strength, suggesting that NS is more effective in activating RPG and RPP. The activation of Ca and Al in RPG plays a positive role in the formation of hydration products, thereby enhancing the material’s compressive strength. However, as the levels of NH and NS increase, the compressive strength and cross-sectional loss rate of the samples do not follow a consistent trend. This implies that the variations in NH and NS concentrations in the marine environment do not have a direct correlation with the strength of MGPC. The changes in compressive strength are primarily related to the activation degree of RPG and silicate minerals. The alkali–silica reaction expansion between RPG and alkaline activators is a key factor influencing the material’s properties, and the extent of this reaction is closely associated with the alkali–silica reaction size of RPG.

### 3.4. XRD

**Figure 5:**
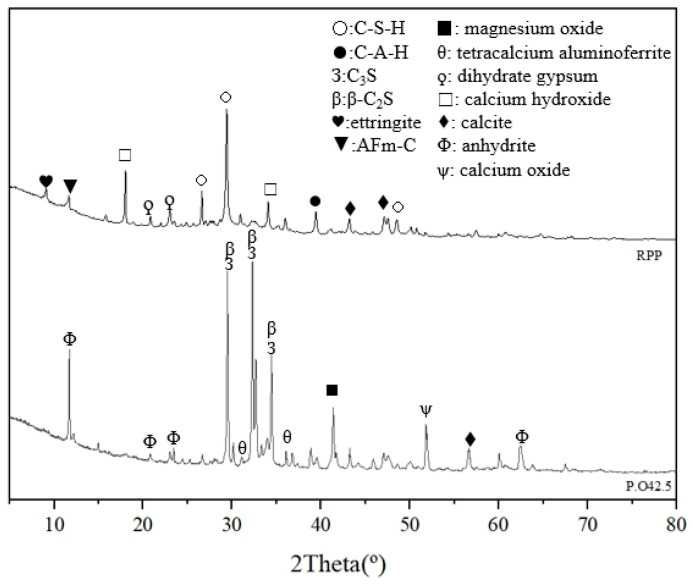
XRD diffraction pattern of P.O 42.5 and RPP. RPP: recycled particles from paste; AFm-C: calcium carboaluminate (CO_3_-AFm).

The XRD diffraction pattern of P.O 42.5 and RPP are shown in Figure 5. By examining the XRD diffraction pattern of P.O 42.5, it is clear that the hydration potential of P.O 42.5 originates from compounds like 3CaO·SiO_2_ (C_3_S), 2CaO·SiO_2_ (β-C_2_S), and C_3_A. Various hydration products, such as calcium silicate hydrate (C-S-H) and 3CaO·Al_2_O_3_·6H_2_O (C-A-H), have also been identified in RPP. These observations, combined with previous findings, confirm the feasibility of using RPP as a recycled construction solid waste.

XRD diffraction pattern of RPP revealed the presence of CO_3_-AFm (3CaO·Al_2_O_3_·CaCO_3_·32H_2_O), MgCO_3_, and CaCO_3_, which are the primary products of cement’s carbon fixation effect. The specific formation mechanisms of CO_3_-AFm are outlined in Equations (3) and (4) [36].

(3) $$3\mathrm{CaO}\cdot {\mathrm{Al}}_{2}O_{3}+3Ca{\left(OH\right)}_{2}+3CO_{2}+8H_{2}O\to CaO\cdot Al_{2}O_{3}\cdot CaCO_{3}\cdot 11H_{2}O$$

(4) $$3(CaO\cdot Al_{2}O_{3})+CaCO_{3}+17H_{2}O\to 3CaO\cdot Al_{2}O_{3}\cdot CaCO_{3}\cdot 11H_{2}O+2(Al_{2}O_{3}\cdot 3H_{2}O)$$

RPP still had some calcium cations that had not formed low solubility CaCO_3_ after the hydration reaction, and the calcium elements still existed in the form of unreacted silicate minerals with the potential for rehydration. Therefore, it is feasible to enhance the strength of the GPC structure by using alkaline activators to further hydrate RPP. 

**Figure 6:**
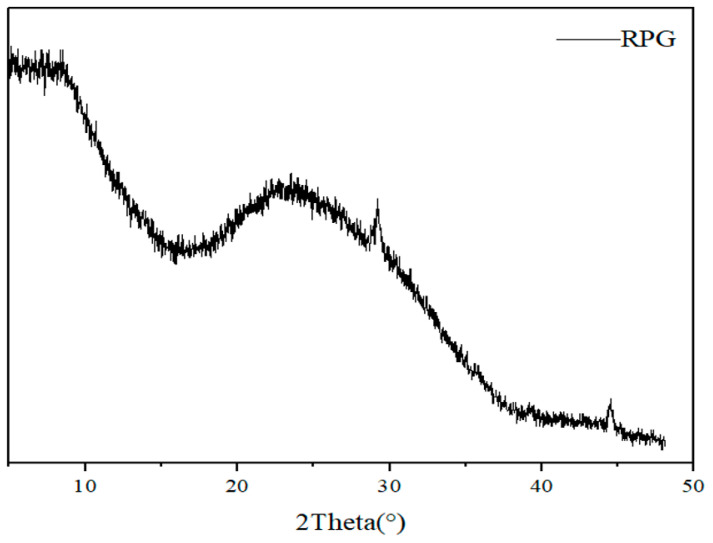
XRD diffraction pattern of RPG. RPG: recycled particles from glass.

The XRD diffraction pattern of RPG, as presented in Figure 6, revealed no detectable crystalline phases, indicating that RPG possesses an amorphous structure. This amorphous nature is the key factor contributing to its high reaction potential [37].

**Figure 7:**
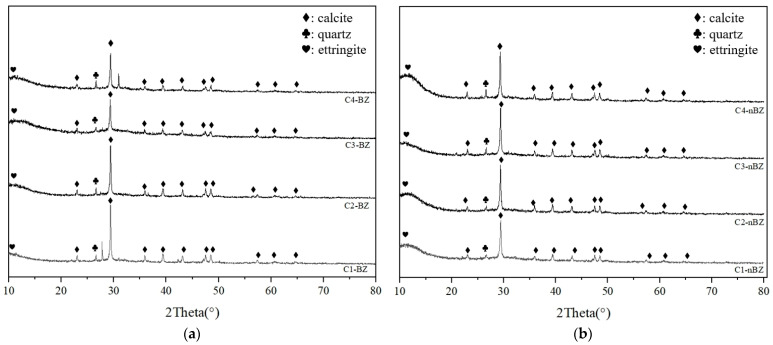

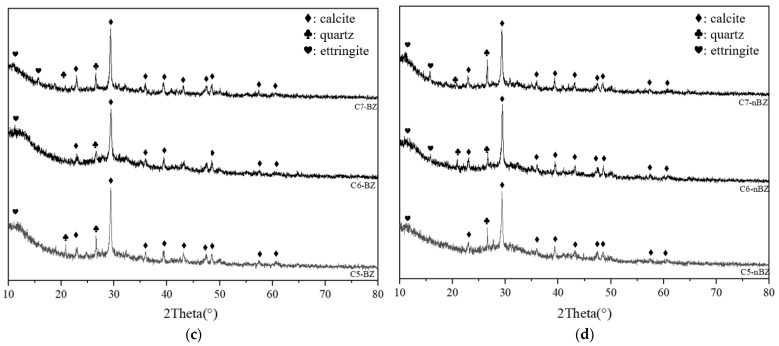
XRD diffraction pattern of MGPC:(**a**) C1–C4 broken zone (BZ); (**b**) C1–C4 no broken zone (nBZ); (**c**) C5–C7 broken zone (BZ); (**d**) C5–C7 no broken zone (nBZ); BZ: broken zone; nBZ: no broken zone.

The XRD diffraction pattern of seven groups of MGPC is shown in Figure 7. By observing the XRD diffraction pattern of the seven scheme samples, it can be seen that CaCO_3_ has the highest diffraction intensity, which indicates that the main product after maintenance in a seawater environment is CaCO_3_. Compared with RPP, there are no phases of calcium hydroxide (Ca(OH)_2_, CH) and CO_3_-AFm in the C1 to C7 group samples. It is because in environments rich in Cl^−^ and SO_4_^2−^, CO_3_-AFm are extremely unstable; they will react with Cl^−^ and SO_4_^2−^ in the environment to form Friedel’s salt and AFt, as shown in Equations (5) and (6) [38]. The reason that CH is not detected is that it reacts with CO_2_ in the environment and generates CaCO_3_.

(5) $$3CaO\cdot Al_{2}O_{3}\cdot {\mathrm{CaCO}}_{3}\cdot 11H_{2}{O+\mathrm{Ca}}^{2+}+2{\mathrm{Cl}}^{-}\to 3CaO\cdot Al_{2}O_{3}\cdot {\mathrm{CaCl}}_{2}\cdot 10H_{2}O+CaCO_{3}+H_{2}O$$

(6) $$3CaO\cdot Al_{2}O_{3}\cdot {\mathrm{CaCO}}_{3}\cdot 11H_{2}{O+\mathrm{Ca}}^{2+}+3{{\mathrm{SO}}_{4}}^{2-}\to 3CaO\cdot Al_{2}O_{3}\cdot {\mathrm{CaSO}}_{4}\cdot 32H_{2}O+CaCO_{3}$$

### 3.5. EDS-SEM Results

**Figure 8:**
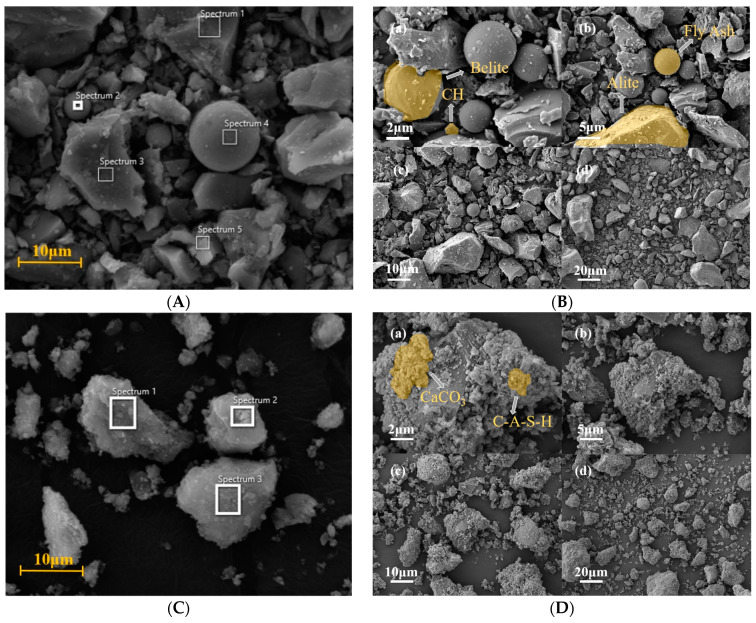
SEM graphic and EDS spectrum selection for P.O 42.5 and RPP: (**A**) EDS spectrum selection for P.O42.5; (**B**) SEM graphic for P.O 42.5; (**C**) EDS spectrum selection for RPP; (**D**) SEM graphic for RPP.

The SEM analysis and EDS spectral results for P.O 42.5 and RPP are presented in Figure 8 and Table 6. Based on the particle shape and elemental composition at each spectrum in Figure 8A and Table 6, it has been determined that Spectra 3 corresponds to Alite, Spectrum 2 to Belite, and Spectrum 4 to fly ash. These materials serve as raw materials, including C_3_S, β-C_2_S, C_3_A, and 4CaO·Al_2_O_3_·Fe_2_O_3_ (C_4_AF), for the hydration reaction of cement. C_3_A initially undergoes hydration, forming C-A-S-H with CaO, and then quickly reacts with CaSO4·2H2O to form AFt, which contributes less to compressive strength compared to C3S. C3S also undergoes hydration shortly after C3A begins to react, becoming the main source of early strength development.

Based on the particle shape and elemental composition at each spectrum in Figure 8C and Table 6, it has been determined that the main phases in spectra 1, 2, and 3 are hydration products such as CH, C-S-H, AFt, and CaCO_3_, indicating good hydration performance of the cement used in this study. Additionally, newly generated CaCO_3_ is found on the outer side of CH and C-S-H crystals, predominantly in the form of calcite. CaCO_3_ forms through the carbonation of cement in contact with CO_2_ in the air during hydration. This occurs when CO_2_ dissolves in the water within cement pores, producing free CO_3_^2−^ that reacts with Ca^2+^ from silicate minerals to create precipitates. This process reduces Ca^2+^ concentration, allowing silicate minerals to dissolve more Ca^2+^.

Previous studies indicate that C_3_S and β-C_2_S exhibit higher reactivity with CO_2_, whereas C_3_A shows lower reactivity, and C_4_AF has negligible reactivity with CO_2_ [39,40]. The reaction equations of silicate minerals and CH with CO_2_ are shown in Equations (7) and (8) [41]. During MGPC mixing, hydration products begin to react with CO_2_ simultaneously with RPP hydration. When C-S-H and CH contact CO_2_, C-S-H decomposes into silica gel and CaCO_3_, leading to a loss of its cementitious properties, while CH reacts with CO_2_ to form CaCO_3_, which reduces the pH in the MGPC. These reactions are illustrated in Equations (9) and (10) [42,43].

(7) $$3CaO\cdot \mathrm{Si}O_{2}+yH_{2}O+\left(3-x\right)CO_{2}\to CaO\cdot \mathrm{Si}O_{2}\cdot yH_{2}O+\left(3-x\right)CaCO_{3}$$

(8) $$2CaO\cdot \mathrm{Si}O_{2}+yH_{2}O+\left(2-x\right)CO_{2}\to CaO\cdot \mathrm{Si}O_{2}\cdot yH_{2}O+\left(2-x\right)CaCO_{3}$$

(9) $$Ca{\left(OH\right)}_{2}+CO_{2}\to CaCO_{3}+H_{2}O$$

(10) $$C-S-H+CO_{2}\to CaCO_{3}+\mathrm{Si}O_{2}+H_{2}O$$

The EDS analysis results shown in Table 6 reveal notable differences between the P.O 42.5 group and the RPP group. It was observed that the carbon content in the RPP group is higher than that in the P.O 42.5 group. This increase is attributed to the involvement of CO_2_ during the mixing and curing stages, where CO_2_ reacts to form solid CaCO_3_, indicating a primary mechanism for carbon fixation in MGPC.

Comparing the Ca and Si content in P.O 42.5 and RPP, the results indicate that the Ca/Si atomic ratio in RPP is higher than in P.O 42.5. This elevated ratio enhances the RPP’s ability to absorb more CO_2_ during the hydration process. The ability to fix CO_2_ through mineral carbonation can be further evaluated by analyzing the presence of Ca and Mg elements. CO_2_ mineral fixation is accomplished by converting CO_2_ into stable inorganic carbonates via reactions with alkaline earth metal cations (Ca^2+^/Mg^2+^) found in natural minerals or solid waste, thereby achieving carbon sequestration [11].

**Figure 9:**
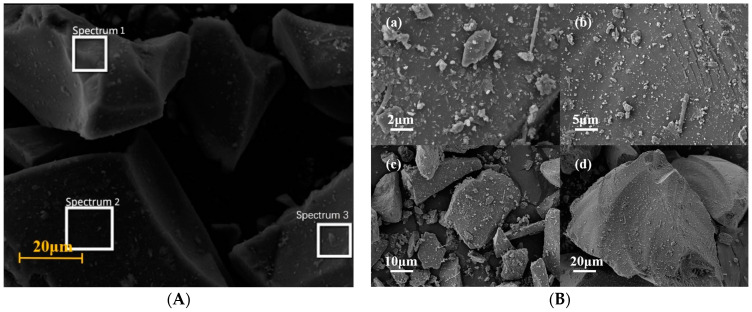
SEM graphic and EDS spectrum selection for RPG: (**A**) EDS spectrum selection; (**B**) SEM graphic.

Figure 9 and Table 7 present the SEM test results and EDS analysis for RPG. RPG exhibits a sharp shape with a cracked surface. The cracked surface facilitates the adhesion of cement to the gel formed after hydration. Consequently, the hydrated gel can form a thicker paste layer on the RPG [44]. Additionally, the Ca and Fe content in RPG was found to be below 15%, which enhances the compressive strength of concrete mixed with RPG, as lower levels of calcium and iron contribute to improved structural integrity [45]. Furthermore, the presence of Al in RPG facilitates the formation of C-A-S-H in hydration products, thereby positively influencing the strength development of MGPC materials.

**Figure 10:**
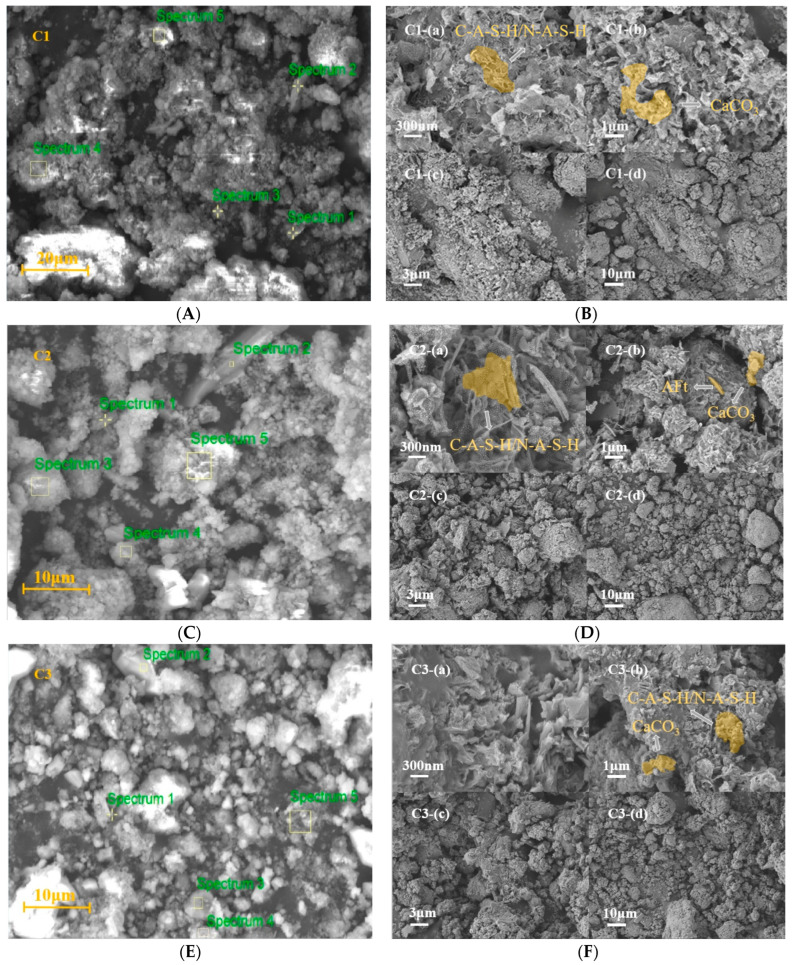

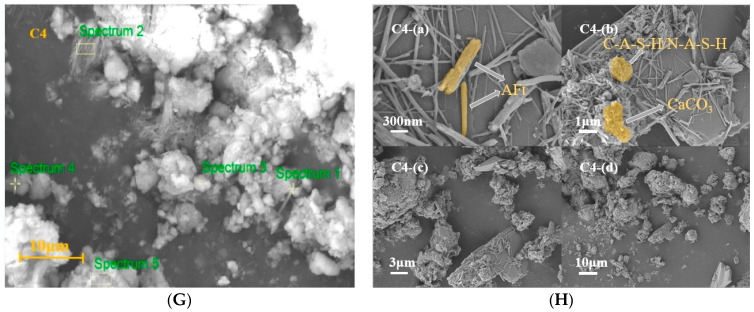
SEM graphic and EDS spectrum selection for C1-C4: (**A**) EDS spectrum selection for C1; (**B**) SEM graphic for C1; (**C**) EDS spectrum selection for C2; (**D**) SEM graphic for C2; (**E**) EDS spectrum selection for C3; (**F**) SEM graphic for C3; (**G**) EDS spectrum selection for C4; (**H**) SEM graphic for C4.

Figure 10 and Table 8 present the SEM test results and EDS spectral analysis of groups C1–C4. The SEM results for groups C1–C3 in Figure 10A–F indicate that the surface of the samples is rough and covered with a significant amount of gelatinous substances. This large amount of gelatinous material is attributed to a violent alkali–silica reaction, resulting in the stacking of multiple phases. The microstructure of MGPC reveals that the RPG surface is covered with newly formed silica gel, while N-A-S-H and C-A-S-H grow outside the RPG in a grid-like formation [46]. CaCO_3_ was found adjacent to the grid-like N-A-S-H and C-A-S-H. The SEM results for group C4 presented in Figure 10H indicate that a large number of needle-shaped crystals are attached to the surface of MGPC. In spectrum 2 of Figure 10G, a large amount of needle-like structures was observed. Based on the elemental composition of group C4 spectrum 2 in Table 8 and the S/Ca atomic ratio in this area, it can be inferred that the primary substance here is AFt. The formation of this structure is due to the initial generation of a large amount of CO_3_-AFm in this area, which subsequently decomposed into CaCO_3_ and AFt upon immersion in seawater.

**Figure 11:**
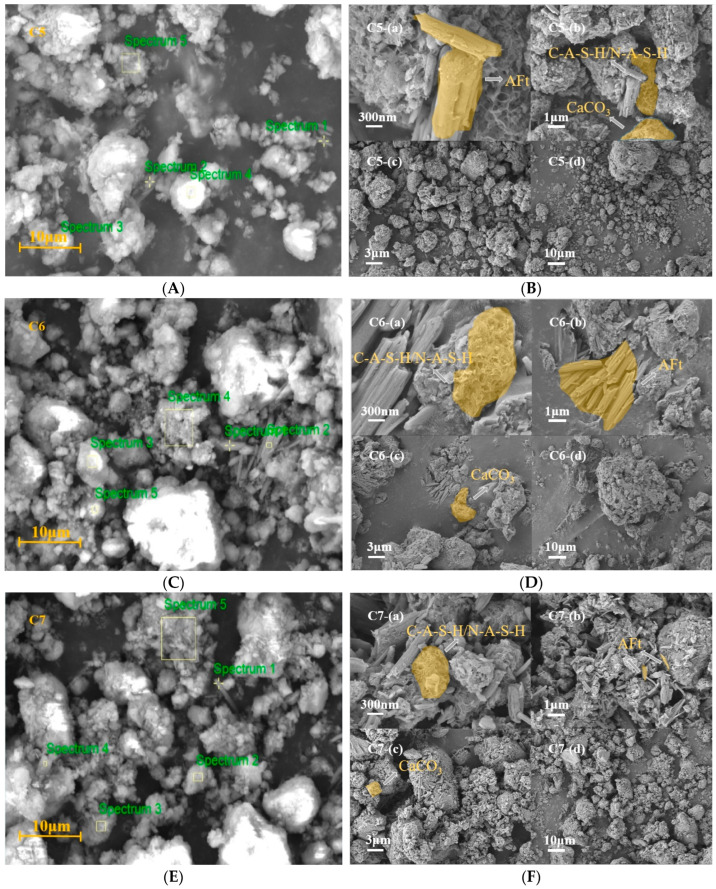
SEM graphic and EDS spectrum selection for C5–C7: (**A**) EDS spectrum selection for C5; (**B**) SEM graphic for C5; (**C**) EDS spectrum selection for C6; (**D**) SEM graphic for C6; (**E**) EDS spectrum selection for C7; (**F**) SEM graphic for C7.

Figure 11 shows the EDS spectra selections and SEM graphics of groups C5 to C7, while Table 9 lists the normalized mass percentages of elements obtained from the EDS tests at different spectrums. Through the analysis of the EDS results and SEM graphics of groups C5 to C7, it can be observed that in the MGPC prepared with NS as an alkaline activator, the surface of the RPG is covered with N-A-S-H, C-A-S-H, CaCO3, and AFt. In Spectrum 4, Figure 11C, fibrous material was observed. Through EDS analysis and previous research findings, it can be determined that AFt was growing in this area. Furthermore, the presence of rough surface materials resulting from intense alkali–silica reactions is also rarely observed in the MGPC prepared with NS as the alkaline activator. Based on the microstructure of the MGPC produced with NS, it can be concluded that the use of NS as an alkaline activator in the environment contributes to the formation of a well-structured MGPC, thereby enhancing its compressive strength.

## 4. Conclusions

This study investigates the preparation of marine geo-polymer cement at room temperature using alkaline activators and recycled construction solid waste (recycled particles from the paste and recycled particles from glass) as substitutes for natural sand and gravel. Under conditions of seawater mixing and curing, the alkaline activators effectively activated elements such as calcium (Ca), aluminum (Al), and silicon (Si) in recycled particles from the paste and recycled particles from glass. This study assessed the compressive strength of the marine geo-polymer cement cubes and measured the mass loss rate and area loss rate after testing to evaluate their strength and toughness. The results indicate that the marine geo-polymer cement cubes containing 10.57% NS exhibited significant compressive strength and good toughness.

The recycled particles from the paste used in this study had undergone carbonization, primarily forming products such as CaCO_3_ and CO_3_-AFm. Using seawater and recycled particles from glass in marine geo-polymer cement preparation facilitated the conversion of CO_3_-AFm in recycled particles from a paste into a more stable form of CaCO_3_, contributing to a reduction in CO_2_ concentration in the air.

By analyzing the proportions of various elements in different mix ratios of marine geo-polymer cement, it was determined that the magnesium (Mg) content is significantly lower than that of calcium (Ca), indicating that the predominant mechanism of CO_2_ mineral fixation in marine geo-polymer cement involves the reaction of Ca^2+^ with dissolved CO_3_^2−^ to form CaCO_3_.

This study demonstrated that alkali metal elements, including sodium (Na), calcium (Ca), and magnesium (Mg), are involved in the hydration process. Most aluminum, silicon, and calcium in construction solid waste are reactivated by alkaline activators, enhancing the formation of aluminum silicate condensation macromolecules. The resulting products, such as N-A-S-H and C-A-S-H, form the main polymer structure of marine geo-polymer cement and are significant sources of strength for the concrete. The fundamental components of these phases are silicon-oxygen tetrahedra (SiO_4_^4−^) and aluminum-oxygen tetrahedra (AlO_4_^5−^). These tetrahedra interconnect to form a complex three-dimensional network by sharing oxygen atoms. The interconnected three-dimensional network created by these tetrahedra forms a large-scale aluminosilicate framework through bridging oxygens (such as Si-O-Si or Al-O-Si bonds). These bridging oxygens serve to connect the silicon and aluminum atoms, creating a continuous structural framework that imparts strength and stability to the system [47,48].

Marine geo-polymer cement exhibits a more compact and low-porosity structure due to the presence of these aluminosilicate networks, which enhances its mechanical properties. Microstructural analysis revealed that the hydration products formed a network-like cementitious matrix covering the recycled particles from glass. The outer layer of larger recycled particles from glass is covered with CaCO_3_, N-A-S-H, and C-A-S-H, contributing to a denser and more compact microstructure. This structure’s advantage is that the hydration products form a protective shell around unreacted recycled particles from glass, preventing alkali–silica reactions [49]. This mechanism helps maintain the stability of the material during use [50].

In the construction industry, using recycled solid waste as cementitious materials can not only reduce the extraction of natural minerals but also effectively reduce the accumulation of building solid waste. Replacing freshwater with seawater can significantly reduce the consumption of freshwater resources. The marine geo-polymer cement developed by this study has both environmental and economic benefits. Through the use of controllable simulation in the design and preparation of construction solid waste, it is found that substances such as Al, Si, and Ca, which are commonly present in construction solid waste, have great value in industrialization, scale, and fine recycling and utilization. However, the alkali silica reaction in this study remains an issue that cannot be ignored. It is necessary to reduce the adverse effects of the alkali–silica reaction on marine geo-polymer cement by using different types of alkaline activators and varying the mix proportions.

## 5. Perspectives

Further research is needed to investigate the effects of varying alkaline activator concentrations on the compressive strength of marine geo-polymer cement in order to identify the most suitable mix proportions. Marine geo-polymer cement materials demonstrate the capability to convert carbonized hydration products into a more stable form of CaCO_3_, indicating their potential for effective carbon fixation. This characteristic enhances the value of marine geo-polymer cement in environmental protection and sustainable development. With scientifically designed mix proportions, these materials can be utilized not only in the construction of buildings or infrastructure within coastal industrial parks but also to significantly reduce CO_2_ concentrations in the environment, thereby mitigating the effects of greenhouse gases. Furthermore, the application of such materials contributes to the transition of the construction industry towards low-carbon and environmentally friendly practices, providing a viable solution to address global climate change.

## Figures and Tables

**Table 1:** Detailed information on alkaline activators.

Alkaline Activators	Specification	Molar Concentration (mol/L)	Appearance
Sodium hydroxide(NaOH)	AR	51.2	White particles
Sodium silicate liquid(Na_2_O·3.3SiO_2_)	CP	2.43	Transparent liquid

AR: Analytical Reagent. CP: Chemically Pure. NaOH: Shanghai Zhanyun Chemical Co., Ltd., Shanghai, China; Na_2_O·3.3SiO_2_: Shandong Yousuo Chemical Technology Co., Ltd., Jinan, China.

**Table 2:** The proportion of each component in MGPC.

Name of Alkaline Activators	Group	Mix	Alkaline Activator (%)	Mass Ratio	L/S
NaOH	C1	RPP:RPG:NH:SW	10.90	1:0.05:0.18:0.42	0.57
	C2	RPP:RPG:NH:SW	10.52	1:0.05:0.18:0.48	0.63
	C3	RPP:RPG:NH:SW	7.27	1:0.05:0.12:0.48	0.57
	C4	RPP:RPG:NH:SW	3.77	1:0.05:0.06:0.48	0.51
Na_2_O·3.3SiO_2_	C5	RPP:RPG:NS:SW	10.52	1:0.05:0.18:0.48	0.63
	C6	RPP:RPG:NS:SW	7.27	1:0.05:0.12:0.48	0.57
	C7	RPP:RPG:NS:SW	3.77	1:0.05:0.06:0.48	0.51

RPP: recycled particles from paste; RPG: recycled particles from glass; NH: NaOH; NS: Na_2_O·3.3SiO_2_; SW: seawater; L/S: (seawater + alkaline activator)/(RPC + RPG).

**Table 3:** Experiment and Equipment.

Experiment	Equipment
Particle Size Analysis	Mastersizer 2000 Tester
Uniaxial Compression Test	YAW-2000 Microcomputer Controlled Electro-Hydraulic Servo Press Tester
X-ray Diffraction	Ultima IV Type Multifunctional Horizontal X-ray Diffractometer
Energy Dispersive Spectroscopy	AZtec X-MaxN 80 Energy Spectrometer
Scanning Electron Microscopy	Tescan Mira 3 XH Field Emission High-Resolution Scanning Electron Microscopy

Mastersizer 2000 Tester: Malvern Instruments Limited, Worcestershire, UK; YAW-2000 Microcomputer Controlled Electro-Hydraulic Servo Press Tester, Wuhan YAW Technology Co., Ltd., Wuhan, China; Ultima IV Type Multifunctional Horizontal X-ray Diffractometer, Rigaku Corporation, Tokyo, Japan; AZtec X-MaxN 80 Energy Spectrometer, Oxford Instruments, Oxfordshire, UK; Tescan Mira 3 XH Field Emission High-Resolution Scanning Electron Microscopy, Tescan Orsay Holding, Brno, Czech Republic.

**Table 4:** Proportion of particle sizes in RPP and RPG.

Type	The Maximum Particle Size Smaller than This Percentage (μm)						
	<10%	<30%	<50%	<60%	<100%	C_u_	C_c_
RPP	2.21	4.95	8.56	11.03	79.9	5.21	1.43
RPG	12.16	42.62	55.72	61.72	300	5.08	2.42

RPP: recycled particles from paste; RPG: recycled particles from glass.

**Table 5:** Mass loss rate and cross-sectional loss rate of MGPC cubes under different mix proportions.

Group	*Ω*_m_ (Average)	*Ω*_a_ (Average)
C1	0.025	0.039
C2	0.076	0.506
C3	0.007	0.022
C4	0.035	0.152
C5	0.008	0.022
C6	0.023	0.188
C7	0.005	0.051

**Table 6:** EDS spectral analysis results of PO42.5.

	Spectrum	Si/Ca	Al/Ca	S/Ca	Mg/Ca	C(%)	O(%)
P.O 42.5	Spectrum 2	1.26	2.91	0.04	0.21	6.57	38.52
	Spectrum 3	3.75	0.28	0.09	0.10	0.00	21.67
	Spectrum 4	6.20	5.76	0.00	0.06	3.59	41.39
	Mean	2.76	2.41	0.13	0.24	3.11	31.60
RPP	Mean	0.14	0.06	0.02	0.06	14.50	35.80

RPP: recycled particles from paste.

**Table 7:** EDS spectral analysis results of RPG: Standardized quality percentage [%].

Spectrum	C	O	Na	Mg	Al	Si	K	Ca	Cr	Fe	Cu	Ba
Mean	4.59	39.14	7.76	0.64	1.41	28.39	0.86	11.84	0.65	2.02	1.80	0.90

**Table 8:** EDS spectral analysis results of C1–C4.

	Spectrum	Si/Ca	Al/Ca	S/Ca	Mg/Ca	Na/Ca	C(%)	O(%)	Cl(%)
C1	Mean	0.96	0.26	0.01	0.09	0.18	7.91	47.24	0.57
C2	Mean	0.41	0.07	0.00	0.02	0.07	10.73	48.74	0.21
C3	Mean	0.05	0.05	0.01	0.03	0.04	22.06	35.55	2.92
C4	Spectrum 2	0.12	0.19	0.20	0.01	0.04	47.15	32.91	0.23
	Mean	0.34	0.09	0.02	0.03	0.05	24.33	45.27	0.75

**Table 9:** EDS spectral analysis results of C5–C7.

	Spectrum	Si/Ca	Al/Ca	S/Ca	Mg/Ca	Na/Ca	C(%)	O(%)	Cl(%)
C5	Mean	0.22	0.25	0.27	0.03	0.03	15.57	25.7	0.62
C6	Spectrum 2	0.06	0.18	0.22	0.01	0.01	5.66	28.1	0.69
	Mean	0.14	0.14	0.11	0.02	0.03	12.03	25.32	0.57
C7	Mean	0.40	0.20	0.06	0.03	0.02	19.72	22.81	0.6

## Data Availability

The data is not disclosed due to privacy concerns to protect the confidentiality of the participants, in accordance with ethical guidelines and institutional policies.
